# Comparison of the Optical Properties of Graphene and Alkyl-terminated Si and Ge Quantum Dots

**DOI:** 10.1038/s41598-017-12872-9

**Published:** 2017-10-31

**Authors:** Chris de Weerd, Yonghun Shin, Emanuele Marino, Joosung Kim, Hyoyoung Lee, Saba Saeed, Tom Gregorkiewicz

**Affiliations:** 1 Institute of Physics, University of Amsterdam, Science Park 904, 1098 XH Amsterdam, The Netherlands; 2 Centre for Integrated Nanostructure Physics, Institute of Basic Science, Department of Chemistry, Sungkyunkwan University, 2066 Seoburo, Jangan-gu, Suwon, Gyeonggi-do 16419 Republic of Korea; 3 Present Address: Centre for Integrated Nanostructure Physics, Institute of Basic Science, Department of Energy Science, Sungkyunkwan University, 2066 Seoburo, Jangan-gu, Suwon, Gyeonggi-do 16419 Republic of Korea; 4 Department of Physics, The Women University, Kutchery Campus, L.M.Q. Road, Multan, Pakistan

## Abstract

Semiconductor quantum dots are widely investigated due to their size dependent energy structure. In particular, colloidal quantum dots represent a promising nanomaterial for optoelectronic devices, such as photodetectors and solar cells, but also luminescent markers for biotechnology, among other applications. Ideal materials for these applications should feature efficient radiative recombination and absorption transitions, altogether with spectral tunability over a wide range. Group IV semiconductor quantum dots can fulfill these requirements and serve as an alternative to the commonly used direct bandgap materials containing toxic and/or rare elements. Here, we present optical properties of butyl-terminated Si and Ge quantum dots and compare them to those of graphene quantum dots, finding them remarkably similar. We investigate their time-resolved photoluminescence emission as well as the photoluminescence excitation and linear absorption spectra. We contemplate that their emission characteristics indicate a (semi-) resonant activation of the emitting channel; the photoluminescence excitation shows characteristics similar to those of a molecule. The optical density is consistent with band-to-band absorption processes originating from core-related states. Hence, these observations strongly indicate a different microscopic origin for absorption and radiative recombination in the three investigated quantum dot systems.

## Introduction

Quantum dots (QDs) received much attention due to their interesting optical properties related to quantum confinement induced effects^1–3^. Specifically, as the QD size decreases and approaches the Bohr radius of the material, the quantum confinement (QC) sets in, modifying the wave function of the electrons and holes^4–7^. Consequently, the energy band structure is affected and in the case of semiconductor QDs, the bandgap energy increases. In addition, as a result of the *so-called* phonon bottleneck effect, the relaxation of hot free carriers through phonon emission can be slowed down by orders of magnitude^8,9^. Semiconductor colloidal QDs offer interesting possibilities for (low-cost) photovoltaic solutions^10,11^ and they can be easily integrated into light emitting devices as emitters^12–17^. For these applications, materials with spectral tunability, high radiative rate and efficient emission and absorption are desired. Group IV semiconductors (i.e. Si, Ge and C) are characterized by large abundance, chemical and structural stability and non-toxicity. However, their optical properties are inferior to those of II-VI and III-V semiconductor QDs (e.g. CdSe, CdTe, PbSe and InAs). Even though Si QDs have been reported to exhibit high photoluminescence quantum yields (PL QYs)^18,19^, their external quantum efficiency (EQE, i.e. the ratio between the number of incident photons and the collected photocarriers) is often not sufficient for implementation in optical devices^20^. It is possible to enhance the optical activity of Si QDs by passivating the surface with oxygen, hydrogen or carbon^21–23^. In particular, capping the surface with aliphatic carbon chains has been reported to have a dramatic effect on the optical properties of group IV semiconductor QDs. For example, 1-octadecene capped Ge QDs show tunable PL in the infrared region with a QY of ~8%^24^ where values typical for ‘bare’ Ge QDs are generally around 1%. Furthermore, tunable PL in the visible region with lifetimes of the order of nanoseconds has been reported for free-standing alkyl-terminated Si QDs^25–27^, which differs greatly from the infrared emission with µs lifetimes typically observed for oxygen passivated Si QDs. In addition, carbon QDs have raised interest due to their high PL QYs, large absorptivity, fast radiative decay and tunable visible PL^28–34^. It was also shown that their bandgap can be modified by changing the size and the surface chemistry which subsequently determine their optical properties^35–37^. Although much research focused on explaining the origin of the PL and absorption mechanism in alkyl-terminated Si and Ge QDs^38^ and in GQDs^39,40^, comparative studies on these materials are lacking. In this respect, we investigate the optical properties of butyl-terminated Si (C-Si) and Ge QDs (C-Ge) and compare them to those of GQDs.

## Synthesis

The C-Si and C-Ge QDs were synthesized via a wet chemical method^26,27,41–43^ adapted from Kauzlarich *et al* ^44^. Briefly; magnesium silicide (Mg_2_Si) was oxidized with bromine (Br_2_) in refluxing n-octane during 60 hours. Bromo-octanes which form as a major side product are removed by efficient evaporation and fresh n-octane is added to the reaction. Formed bromine-terminated Si-QDs were subsequently passivated using n-butyllithium, replacing the bromine and resulting in n-butyl-terminated Si QDs. To prepare butyl-terminated Ge QDs, Mg_2_Ge was used instead of Mg_2_Si while the same method and remaining materials were handled as described for preparing the butyl-terminated Si QDs. Even though several steps are performed to remove any unreacted bromine from the samples, our energy dispersive X-ray (EDX) analysis reveals a majority of residual Br nanoparticles (see Fig. S1 in the Supplementary Information). Other than Br, no evidence of any remaining side product(s) such as an excess of C or Mg were observed.

According to Fourier Transform Infrared Spectroscopy (FTIR) analysis performed on n-butyl-terminated Si nanoparticles prepared in n-octane^45^, CH_2_ and CH_3_ groups are also present on their surface protecting the Si core against oxidation because the Si-O bond is weak in comparison. Furthermore, brominated octyl species have been observed on the surface of these C-Si QDs as a consequence of the reaction solvent bromination^42^. The GQDs were prepared by the oxidative cleavage of graphene oxides (GOs) to introduce hydroxyl groups in the dots. We used ultrasonication (sono-oxidation) to prepare the GQDs from GOs. After filtration, the GQDs solutions were purified (see also the Supplementary Information for further details). The GQDs have a size distribution of a few tens of nanometers (see Fig. S2 in the Supplementary Information) and according to XPS the GQDs are functionalized with hydroxyl, graphitic carbon, oxygenated carbon, and carboxylic acid groups^46^. All QDs used in this study were dispersed in UV-grade ethanol.

## Results

### Optical characterization

**Figure 1:**
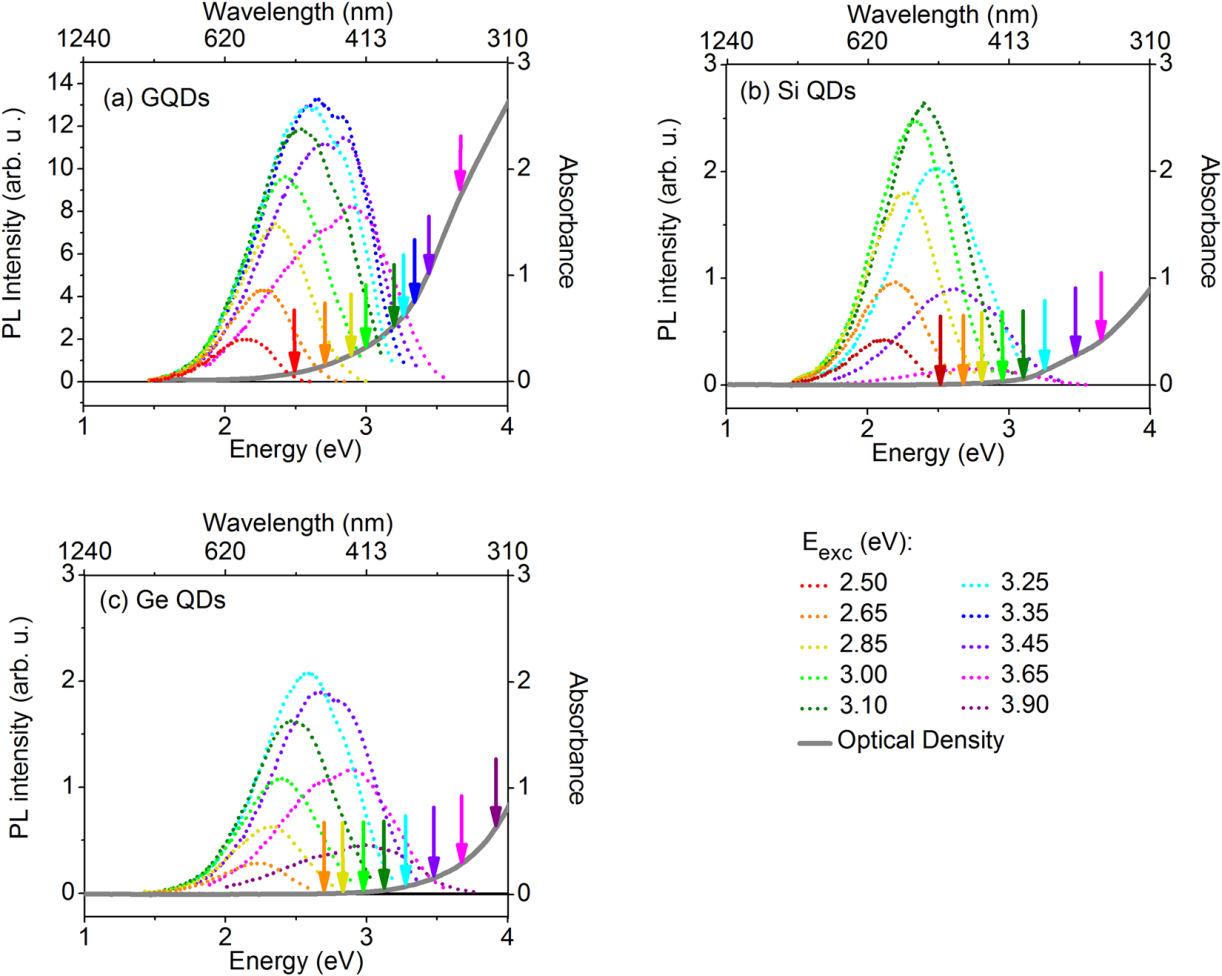
Photoluminescence excitation and optical density. Tunable PL emission (left axis) obtained for different excitation energies ranging between 2.5–3.9 eV for (**a**) GQDS and butyl-terminated (**b**) Si and (**c**) Ge QDs. In all graphs, the optical density is indicated by the gray solid line (right axis). The colored arrows indicate the different excitation energies.

The QDs have been optically characterized and their absorbance and PL spectra at different excitation energies are compared in Fig. 1. The arrows indicate the actual excitation energy along the absorption spectrum, in the same color as the emission spectra. All samples show tunable, visible, blue-green PL, where the PL intensity is gradually increasing and decreasing with excitation energy. The optical density is steadily increasing with energy and does not exhibit an excitonic line at the onset, as expected for indirect bandgap semiconductor QDs.

**Figure 2:**
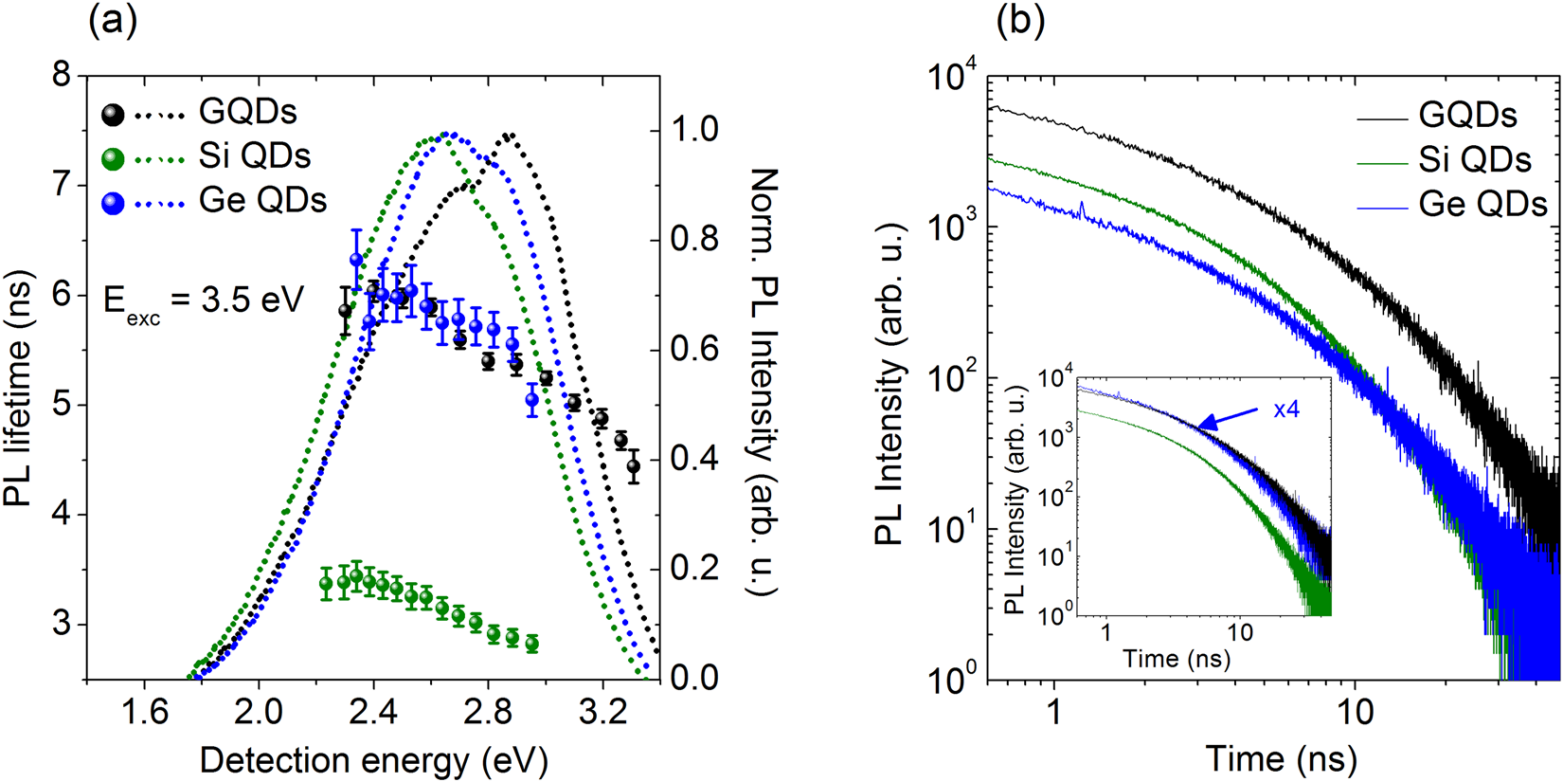
Time-resolved photoluminescence. (**a**) The nanosecond PL lifetimes for the GQDs (black) and C-Si (green) and C-Ge (blue) QDS, as a function of detection energy (2.2–3.3 eV). The excitation energy is 3.5 eV. As expected, due to QC, a decreasing trend of the PL lifetime is observed for increasing detection energy for all three samples. The normalized PL spectra for the same excitation energy (3.5 eV) are indicated by the dotted lines (same colors). (**b**) Time-resolved PL signal at E_det_ = 2.8 eV. The inset shows the transient of the C-Ge QDs multiplied by 4, to illustrate the similarity in decay with the GQDs.

In order to investigate the (radiative) recombination characteristics, the time-resolved PL signal was recorded for all three samples, using an excitation energy of 3.5 eV. The PL lifetimes are in the nanosecond range, as is shown in Fig. 2a, and comparable (5–6 ns, black and blue respectively) for the GQDs and C-Ge QDs, and 3–3.5 ns for the C-Si QDs. In the same graph, the normalized PL spectra are included (dotted lines) for the same excitation energy of 3.5 eV. It is remarkable how similar the PL spectra for the different QDs are, the maxima being at roughly the same position. Time-resolved PL transients detected at 2.8 eV for the three types of QDs investigated here, are shown in Fig. 2b. The inset remarks the similarity between the C-Ge and GQDs. While the total decay had to be fitted with a contribution of three exponents in the range of, respectively, ps, ns and tens of ns, in all cases the nanosecond component dominates (i.e. its amplitude is at least a factor 100 larger than the other two).

**Figure 3:**
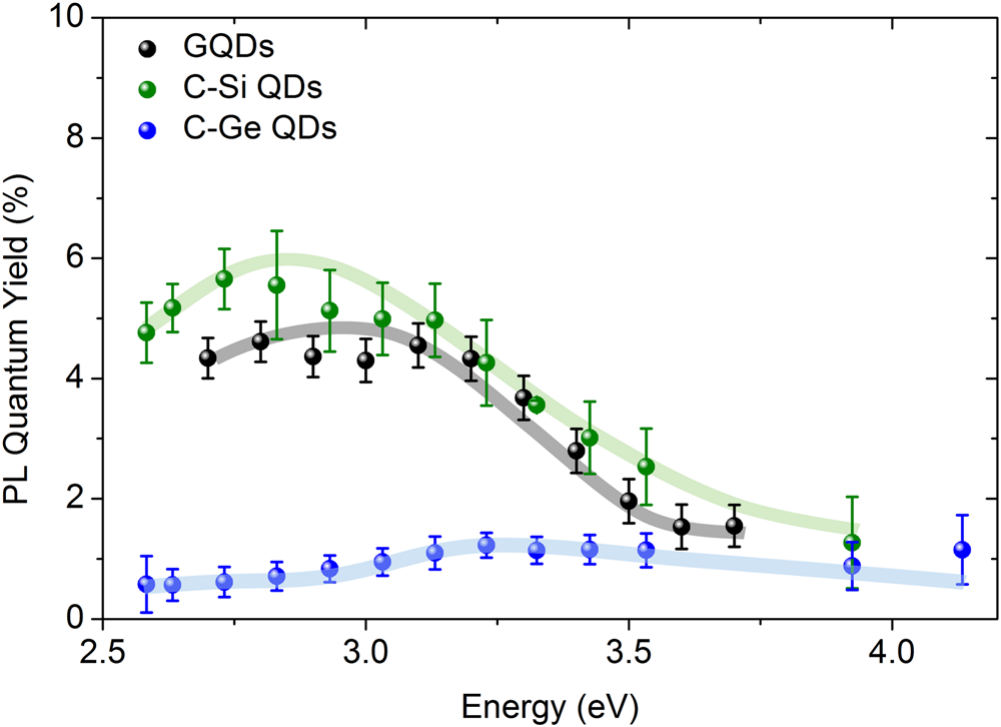
Photoluminescence quantum yield. The absolute PL QY as a function of energy for the GQDs (black), C-Si (green) and C-Ge (blue) QDs. Where a QY of ~5.5% is observed for the GQDs and the C-Si QDs, that of the C-Ge QDs is much lower (~1%). All data points follow the same increasing and decreasing trend with excitation energy. The error bars represent the statistical error as caused by fluctuations of the lamp intensity used as excitation source.

Finally, Fig. 3 shows the absolute PL QY as a function of excitation energy, for all three samples (same colors as Fig. 2). A QY of ~5.5% is measured for the GQDs and C-Si QDs, where a lower value of ~1% was measured for C-Ge QDs, in accordance with values reported in literature^47–50^. The QY dependence on excitation energy shows in all three cases a maximum, for the GQDs and C-Si QDs around 2.8–3 eV and for the C-Ge QDs around 3.2 eV.

## Discussion

The observed optical properties of all three samples are very similar. Their optical density shows a gradual increase with excitation energy, as is expected for an ensemble of (indirect bandgap) QDs. Band-to-band absorption by core states is responsible here, governed by the increase in the density of states further away from the band edges. For the GQDs, the onset in absorption takes place at lower energies, indicating a smaller bandgap. In contrast, the PLE characteristics indicate the presence of a variety/ensemble of emitters that are excited resonantly, giving rise to the increasing and decreasing trend of the PL maximum (with increasing excitation energy) as is typically observed for molecules. In our materials, this can be described by the presence of surface moieties introducing localized states on the QD surface. A surface passivating molecule can, in general, have a boosting or quenching effect on the PL of a QD, depending on the position of the surface states with respect to the QD bandgap. This has been observed for CdTe and CdSe QDs capped with thiol groups^51^ and can be explained by the position of the top of the valence band of the QD with respect to the HOMO/LUMO energy levels of the molecule. If the HOMO (LUMO) level is placed above (below) the top (bottom) of the valence (conduction) band of the QD, this can cause trapping of a hole (electron) upon excitation of an electron-hole pair. The segregation of one carrier species on localized states on the surface of a QD will decrease the electron-hole wave functions overlap, resulting in a strongly reduced radiative efficiency. As the bandgap energy of the QD increases with decreasing size, the efficiency of carrier trapping on the surface may increase, therefore decreasing the QD PL intensity. This is clearly in contrast with what is expected for a QD ensemble, where the QD is the only emitter. In that case, the resulting PLE should follow an ongoing increase in PL intensity upon increasing excitation energy.

We find that the optical density behaves as is expected for a QD ensemble with a size distribution, whereas the PL/PLE does not, indicating that the origin of the two processes differs at the microscopic level. Another feature that should be noted, is the appearance of a double-peak in the PL spectra at high (3.45–3.9 eV) excitation energies for the C-Ge and GQDs. In GQDs, the origin of a double-peak in the PLE is explained by the presence of conjugated π-domains, where the π-π (HOMO-LUMO) and σ-π transitions give rise to the distinctive PL peaks^39^.

As for the PL decay characteristics, all the QD materials here investigated show a similar behavior, featuring nanosecond lifetimes, which are too fast to originate from the core-related (excitonic) emission, as generally observed and expected for indirect bandgap semiconductor QDs. This is, however, consistent with the observations for the PLE and therefore related to the surface states.

We find that the absolute QY of all samples is quite low (<6%). This is explained by the presence of a strong non-radiative recombination channel and/or energy transfer, which is favored over radiative recombination and subsequently quenching the PL QY^52,53^. The presence of non-radiative channels influences the (mono)exponential decay such that it becomes multi- or non-exponential, in agreement with our observations for the time-resolved PL.

Indeed, it is striking we find Br nanoparticles in the C-Si and C-Ge QD samples. However, Br nanoparticles are not optically active. Moreover, the observed excitation dependent PL emission, agrees very well with that of alkyl terminated Si QDs^25–27^, and the measured optical density is characteristic of indirect bandgap semiconductor particles. Therefore, this leads us to conclude that the observed emission and absorption spectra originate most probably from the C-Si or C-Ge QDs.

## Conclusion

In all three types of QDs, the features observed for the PL/PLE are strikingly similar, which indicates that the microscopic origin of radiative recombination for C-Si and C-Ge QDs and GQDs is similar. The emission characteristics indicate (semi-) resonant activation of the emitting channel, since the PL resembles emission characteristics of a molecule. The absorption characteristics are identical and can be identified with band-to-band absorption processes in the QD core. Based on these observations, we postulate that the presence of carbon moieties on the surface is responsible for the similar PL mechanism observed in all three materials.

## Methods

### Optical density

The optical density was measured in a LAMBDA 950 UV/Vis/NIR spectrophotometer (PerkinElmer). A combination of a tungsten-halogen and deuterium lamp is used together with a PMT and Peltier cooled PbS detector, to provide a detection range of E_det_ = 5.6 − 0.4 eV. The sample and solvent measurements have been performed separately and afterwards subtracted from each other.

### Photoluminescence spectroscopy and quantum yield

The PL spectra are recorded by a Jobin Yvon FluoroLog spectrofluorometer (Horiba) equipped with a 450 W xenon lamp (250–700 nm) coupled to a monochromator to provide a selective range of excitation wavelengths. The emission spectra were collected in a right-angle geometry and subsequently scaled for the intensity of the xenon lamp, and corrected for spectral sensitivity. To determine the PL quantum yield the samples are placed in an integrating sphere, using a 150 W xenon lamp coupled to a spectrometer (Solar, MSA-130) as an excitation source. The PL emission and excitation light are scattered diffusively in the integrating sphere and are detected by a CCD (Hamamatsu S10141-1108S).

### Time-resolved photoluminescence spectroscopy

The picosecond PL dynamics have been measured using the frequency-doubled output of a tunable Ti:Sapphire laser system (Chameleon Ultra, Coherent), providing 140 fs pulses at E_exc_ = 2.88 eV. A pulse picker has been employed to reduce the repetition rate from 80 MHz to 8 MHz. The PL emission has been detected using a monochromator (Newport CS260-02) coupled to a PMT (Hamamatsu) providing a total detection range of E_det_ = 6 − 1.6 eV. The overall instrument response function (IRF) is 20–25 ps (FWHM) as measured from a dilute scattering solution (Ludox) at the excitation wavelength. All measurements have been performed at room temperature.

### Transmission electron microscopy (TEM)

The samples were analysed by transmission electron microscopy (TEM) using a JEOL JEM 2010 microscope with a LaB6 thermionic source, operating at an acceleration voltage of 200 kV and equipped with a Gatan multi-scan digital camera.

## Electronic supplementary material

Supplementary Information
