# Supplementary material for: Comparison of the Optical Properties of Graphene and Alkyl-terminated Si and Ge Quantum Dots
Source: Sci Rep. 2017 Oct 31;7:14463. doi: 10.1038/s41598-017-12872-9 (PMC5663913; doi:10.1038/s41598-017-12872-9)
Supplement: Supplementary file 1 — Supplementary Information [file 41598_2017_12872_MOESM1_ESM.pdf]

Supplementary Information for:

Comparison of the Optical Properties of Graphene and Alkyl-terminated Si and Ge Quantum Dots

Chris de Weerd[1*], Yonghun Shin[2], Emanuele Marino[1], Joosung Kim[2], Hyoyoung Lee[2], Saba Saeed[1] and Tom Gregorkiewicz[1*]

[1] Institute of Physics, University of Amsterdam, Science Park 904, 1098 XH Amsterdam, The Netherlands.

[2] Center for Smart Molecular Memory, Department of Chemistry and Department of Energy Science, Sungkyunkwan University, 2066 Seoburo, Jangan-gu, Suwon, Gyeonggi-do 440-746, Republic of Korea

[*]Correspondence and requests for materials should be addressed to:

c.deweerd@uva.nl or t.gregorkiewicz@uva.nl

Synthesis methods and additional figures

Synthesis of butyl-terminated Si and Ge QDs

First, the reaction system was made oxygen free by consecutively flushing with argon and applying a vacuum. Then, magnesium silicide (germanide) was added to the reaction vessel. The system was loaded with 500 ml of n-octane which was bubbled with argon previously. Subsequently, 5.2 ml of bromine was carefully added to the mixture and allowed to stir at room temperature for 2 hours after which the mixture was refluxed for 60 hours at 130 °C. During this reaction, the bromo-octanes which form as a major side product are removed by efficient evaporation and fresh n-octane is added to the reaction. The bromine groups were replaced with butyl groups by adding n-butyllithium (5.2 ml) and the whole mixture was left for overnight stirring at room temperature. The excess lithium reagent was quenched by using 30 ml methanol and stirred for 45 mins. The reaction mixture was filtered and washed repeatedly with aqueous hydrochloric acid solution to remove lithium salts. The hexane fraction was subjected to rotatory evaporator to remove any hexane present. The reaction mixture was subjected to column purification using silica column chromatography using hexane and ethylacetate as the mobile phase.

Synthesis of graphene QDs

Graphene oxide (GO, 100 mg) was synthesized by Hummers method. GO film was first individually dispersed in concentrated DMF (100 mL) with oxone (2 g). The mixture solution was sonicated for 1 h. Then, the mixture was transferred to an autoclave (100 ml) and was heated up to 140 °C for 10 h. The resulting product was then cooled to room temperature and was filtered through a 100 nm nano-porous membrane to separate the carbon based materials. The resulting product solution was further dialyzed in a dialysis bag (retained molecular weight: 2000 Da) for 3 days. See also Ref. 46.

Butyl-terminated Ge and Si QDs:

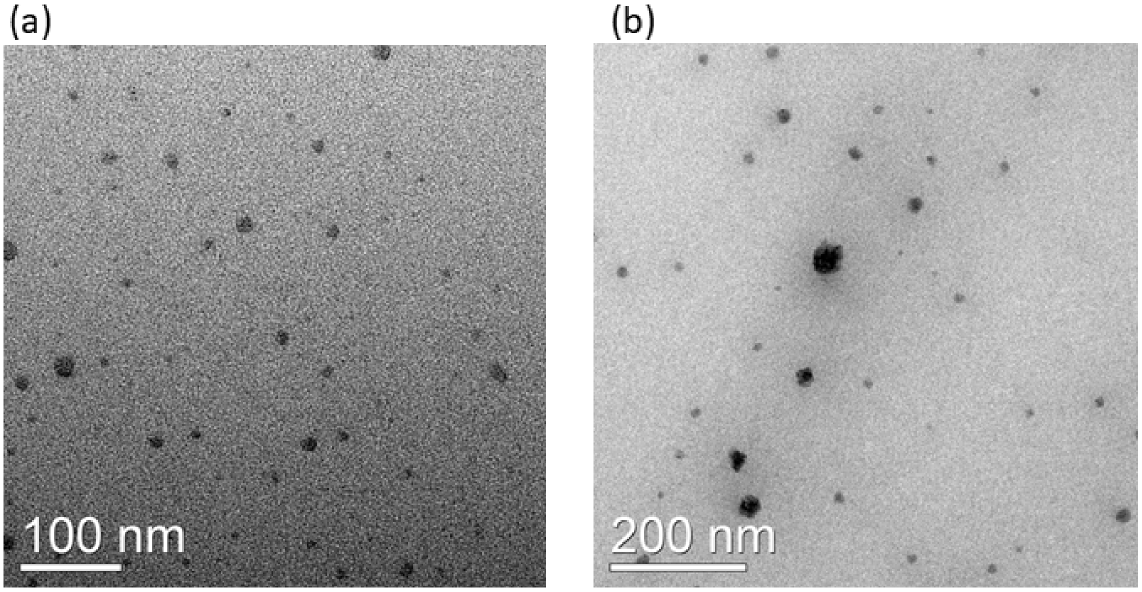

Figure S1: TEM images of residual Br nanoparticles in the C-Ge and C-Si QDs sample. TEM measurements on the C-Ge sample reveal many nanoparticles (a) which consist of pure Br determined by EDX. The same is observed for the C-Si sample (b).

Graphene QDs:

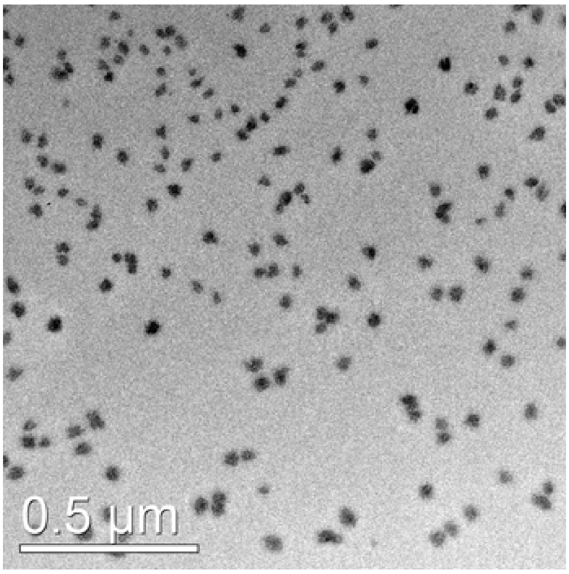

Figure S2: TEM image nanoparticles in the GQDs sample. TEM measurements on the GQDs sample shows nanoparticle with sizes of tens of nanometers.
